# Raising laying hens: housing complexity and genetic strain affect startle reflex amplitude and behavioural response to fear-inducing stimuli

**DOI:** 10.1098/rsos.231075

**Published:** 2024-03-20

**Authors:** Ana K. Rentsch, Alexandra Harlander, Lee Niel, Janice M. Siegford, Tina M. Widowski

**Affiliations:** ^1^ Department of Animal Biosciences, University of Guelph, 50 Stone Road East, Guelph, Ontario N1G 2W1, Canada; ^2^ Campbell Centre for the Study of Animal Welfare, University of Guelph, 50 Stone Road East, Guelph, Ontario N1G 2W1, Canada; ^3^ Department of Population Medicine, University of Guelph, 50 Stone Road East, Guelph, Ontario N1G 2W1, Canada; ^4^ Department of Animal Science, Michigan State University, 474 South Shaw Lane, East Lansing, MI 48824-1225, USA

**Keywords:** laying hen, rearing aviary, fear behaviour, novel object, novel arena, startle reflex

## Abstract

Individual variation in fearfulness can be modified during ontogeny, and high levels of fear can affect animal welfare. We asked whether early-life environmental complexity and genetic strain affect fear behaviour in young laying hens (pullets). Four replicates of brown (B) and white (W) genetic strains (breeds) of layers were each raised in four environmental treatments (housing): conventional cages (*Conv*) and different rearing aviaries with increasing space and complexity (*Low* < *Mid* < *High*). We used a startle reflex test (weeks 4 and 14) to measure startle amplitude and autonomic response (i.e. comb temperature). A combination of novel arena (NA) and novel object (NO) tests was used (week 14) to assess NA exploration and alertness, latency to approach the centre and initial NO avoidance and investigation. Housing × strain affected startle amplitude (B-Conv, B-High < B-Low, B-Mid; B > W; no housing effect in W) but not autonomic response. Fear behaviour was affected by housing (NA exploration, investigation: Conv < Low, Mid, High; NO avoidance: Conv, High < Low, Mid), strain (NA alertness: B > W, NO avoidance: W > B) and their interaction (NA centre approach: B-Conv < all other groups). We present evidence for strain-specific fear responses depending on early experience.

## 1. Introduction

Fear behaviour and fearfulness can help animals cope with threats or risky situations [1]. Many different fields within behavioural sciences (e.g. applied ethology, psychology and behavioural ecology) have established terminology and concepts describing consistent individual variation in behavioural responses to a threat or risk, such as animal personality, behavioural syndrome, temperament or coping style (discussed in Pamela Delarue *et al*. [2]). Most of these concepts assume that individuals fall on a continuum of shy/bold or reactive/proactive and that the emotional and behavioural responses associated with a personality trait are consistent across time and context [2–4]. What all these concepts also have in common is that an individual’s behavioural tendencies are modified by phylogeny, environment and experience during ontogeny [4].

Environmental factors contribute to shaping fear behaviour and perceptions of threatening stimuli for animals in natural conditions [5], as well as in laboratory [6], farmed [7] and companion animals [8]. Across taxa, environmental complexity or degree of exposure to a variety of diverse stimuli has been shown to be particularly critical during ontogeny, influencing the developmental trajectory of fear behaviour and temperament (in mice (*Mus musculus wagneri*) [9], rats (*Rattus norvegicus domestica*) [10], dogs (*Canis familiaris*) [11] and fish (*Gambusia affinis*) [12]). Thus, individual variation in fearfulness can be modified during ontogeny, and if fear is high, it can affect an animal’s welfare [13].

As concern for animal welfare is shaping global farming systems, more attention is being directed towards personality traits, such as fearfulness [3], in farmed species [14,15]. While fear behaviour is adaptive when expressed in the appropriate circumstance, fear is a negative affective state that can lead to distress or injury, if extreme or chronic [14,15]. In large groups of egg-laying hens (*Gallus gallus domesticus*) housed in cage-free aviary housing systems, for example, high levels of fearfulness can increase piling and smothering [16], cause collisions that result in bone fractures [17,18] and predispose hens to injurious behaviour like severe feather pecking [19–21]. Thus, understanding of how ontogeny can affect fearfulness in laying hens can impact the welfare of millions of farmed animals worldwide.

**Figure 1.**
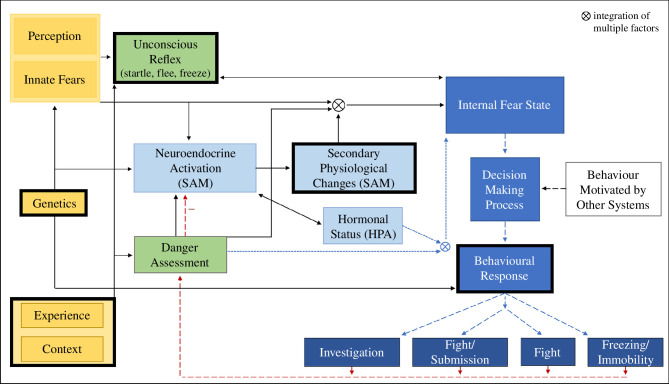
Hypothetical mechanism of an adaptive fear response; amended from Jones, ‘The assessment of fear in the domestic fowl’ [31]. Solid lines represent the initial stage, broken lines the second stage and dotted lines the third stage. Yellow represents factors that are thought to influence the subjective state of fear, green events are in direct response to the stimulus, light blue represents physiological events and dark blue represents the behavioural decision-making tree. Red lines indicate the negative feedback that reduces fear following the appropriate response. Factors and events outlined in black were assessed in this paper. Context includes methodological factors such as testing individually versus in pairs/groups or testing in the home pen versus a testing arena. Other systems influencing the fear response could be reproduction (e.g. protection of young) or foraging (e.g. risk-taking versus food abundance). We propose that genetic effects could be at the level of perception, innate fears, unconscious reflex, physiological response (neuroendocrine activation) and type of behavioural response. Behavioural responses can indicate levels of experienced fear, with ‘investigation’ and potentially ‘immobility’ indicating low levels of fearfulness, while ‘fight’, ‘flight’ and ‘freezing’ indicate high levels of fearfulness. ‘Fight’ and ‘flight’ are active fear behaviours, and ‘freezing’ is an inactive fear behaviour. SAM stands for the sympathomedullary pathway. HPA stands for the hypothalamic–pituitary–adrenal axis.

An animal’s emotionality depends on more than the stimulus type and experience, and it is also influenced by genetic disposition (see figure 1). Domesticated animals are often less fearful of humans compared with their wild ancestral counterparts (e.g. [22]). Different breeds of domesticated animals may also differ in their perception of fear-inducing stimuli (e.g. in dogs (*Canis familiaris*) [23]), and different breeds vary in the level of developmental plasticity of fear behaviour (in sheep (*Ovis aries*) [24], in rats (*Rattus norvegicus domestica*) [25]). Similarly, in the commercial-laying hen, there have been many reports of differences in fear behaviour between different genetic strains and breeds of chicken [26–28]. Commercial-laying hens can be grouped into two phylogenetic groups that were domesticated in different regions of the world [29] and have either brown or white eggshells, usually corresponding to their feather colour [30]. Both phylogenetic groups contain multiple distinct strains, a term used in this paper to refer to specific genetic lines. While white hens have long been thought to be flightier and browns appear more docile [31], differences in fear behaviour between strains might depend on the type of fear-inducing stimulus [28]. In addition, different response patterns were reported: white hens fled (flew up to avoid) from a looming object, whereas brown birds froze [27]. In terms of hen welfare, both the level of fearfulness and the type of fear response should be considered. While ‘freezing’ versus ‘fleeing’ do not necessarily indicate a difference in fearfulness, flightiness can increase the risk of injury through collision, especially in cage-free housing systems [18].

Environmental complexity in the form of the amount and arrangement of physical structures in three-dimensional space has been seen to influence personality traits and physiology in nature (e.g. in the tropics [2]). Structural complexity is used as a type of ‘environmental enrichment’ in captive management systems [32] with the potential to reduce an animal’s emotional reactivity to a stressor (e.g. exposure to a novel object (NO) or novel arena (NA)) (reviewed in Fox *et al*. [6]). While enrichment during adulthood has been shown to decrease fear responses (Nile tilapia (*Oreochromis niloticus* L.) [7], male Japanese quail (*Coturnix coturnix japonica*) [32] and Amazon parrots (*Amazona amazonica*) [33]), the effect appears more potent when provided during development (e.g. [34]). In laying hens, environmental enrichment during early life has been shown to reduce fearfulness in adult laying hens in a human approach and an NO test [15], and cage-reared birds displayed signs of higher levels of fearfulness in NO tests compared with aviary-reared hens [35,36]. However, a concern was raised when aviary-reared birds reacted with flight to a startling stimulus [35], as this could lead to injury through collision [18]. Nonetheless, it has been suggested in multiple reviews that fearfulness in laying hens could be reduced by rearing pullets in complex rearing systems [37,38].

In this paper, we asked whether the degree of early-life housing complexity (minimal, low, moderate or high), genetic strain (brown- versus white-feathered) or their interaction affects fear behaviour (type and intensity) in laying hen pullets in different fear-inducing situations: (i) a startle reflex test, (ii) an NA and (iii) an NO test. Four flocks of Lohman Brown lites and Lohmann Selected Leghorn lites were raised in four environmental treatments consisting of different housing designs: the conventional pullet cage (*Conv*) and three styles of rearing aviaries with increasing space and complexity (*Low* < *Mid* < *High*). In addition, infrared thermography was used to assess sympathetic autonomic response during the startle reflex test in flocks 3 and 4. We hypothesized that increased early-life complexity would decrease indicators of fearfulness in the NA, fearfulness towards the NO, the startle amplitude and the autonomic response to the startle (*Conv* > *Low* > *Mid* > *High*). We predicted that birds with higher levels of fearfulness would explore the NA less, display more agitated behaviour, respond with freezing or fleeing to the NO, take longer to approach it and would have a larger startle amplitude than birds with low levels of fearfulness. We further hypothesized that indicators of fear and fearfulness would differ between brown- and white-feathered birds. However, no directional prediction was made as the literature offers inconsistent evidence of which strain would be more fearful.

## 2. Material and methods

### 2.1. Animals and management

Four consecutive flocks of 1500 Lohman Brown lite (brown) and 1500 Lohman Selected Leghorn lite (white) were hatched at a commercial hatchery (Archer’s Poultry Farm Ltd., Ontario, Canada) and transported to the Arkell Research Station at the University of Guelph. Chicks were vaccinated and beak-treated with an infrared laser at the hatchery. In each flock, one-day-old chicks were divided and placed into one of four rearing environments (see §2.2). Chicks were on an intermittent light schedule, 4 h light:2 h dark, for the first four days, then at 16 h light:8 h dark until five weeks of age (WoA) and finally, 10 h light:14 h dark for the rest of rearing. Light intensity was set to 40 lux on day 1 and was decreased gradually to 10 lux at five WoA. Feed consisted of a standard-laying hen starter diet for the first six weeks and a standard grower diet for weeks 7–17.

### 2.2. Housing

The environmental treatments consisted of four housing designs, all being commercially available pullet rearing systems: conventional pullet cages (*Conv*) and three different styles of rearing aviaries with low (*Low*), moderate (*Mid*) or high (*High*) complexity. Two replicates of each treatment were used per flock, one for each genetic strain (*n* = 8 replicates per housing treatment: 2 strains × 4 flocks). For each strain, there were 4 *Conv* cages holding 30 chicks for the first three weeks that were split between 8 cages for the rest of the rearing. The rearing aviaries differed mainly in the design (space and complexity) of the brooding compartments, which are areas where chicks are confined for the first few weeks of life. *Low* was represented by the Natura Primus system by Big Dutchman (Vechta Germany), a three-tier aviary with three brooding compartments (L: 122 cm × W: 94 cm × H: 50 cm) each holding 115 chicks. Each compartment was furnished with two elevated perches. At three WoA, chicks were split between six compartments. *Mid* was represented by the Natura Filia system (Big Dutchman) with three brooding compartments (L: 147 cm × W: 111.5 cm × H: 90 cm) that held 144 chicks each. This design offered three perches and an elevated platform. The *High*-complexity aviary was represented by two models from different manufacturers: Pullet Portal (PP, five tiers) by Farmer Automatic (Georgia, USA) and NivoVaria (NV, three tiers) by Jansen (VDL Jansen formerly known as Jansen Poultry Equipment, Barneveld, The Netherlands). *High* had 1 large open concept brooding compartment (PP—L: 724 cm × W: 245 cm; NV—L: 726.5 cm × W: 236 cm) with 600 chicks. The brooding compartment was furnished with six perches and an elevated platform. At six WoA, brooding compartments were opened, and all aviary-housed pullets gained access to more perches, multiple tiers, a litter area and ramps. The *Conv* pullets remained in the cages until the end of rearing. Management practices and feeder space per bird were identical in all systems, but stocking density varied; from highest to lowest, *Conv* > *Low* > Mid > *High*. The stocking densities were in compliance with the Canadian Code of Practice [39] for an applicable representation of each housing system (external validity). For illustrations and more detailed descriptions of housing and management, see Rentsch *et al*. [40].

### 2.3. Startle test

**Figure 2.**
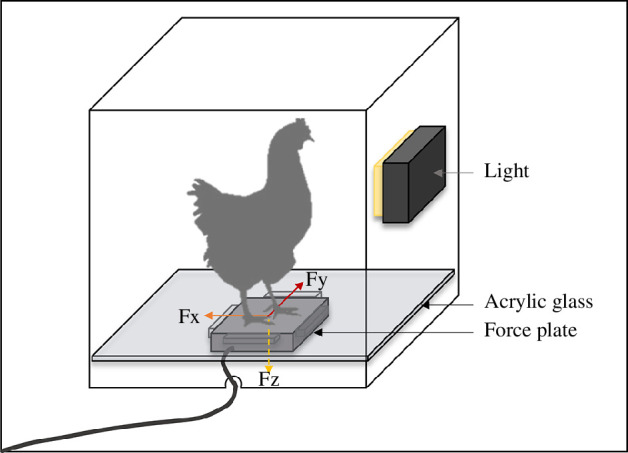
Startle test apparatus. The box (L: 47 cm × W: 47 cm × H: 47 cm) was closed with a lid. A rectangle (W: 11 cm × H: 8 cm) was cut on one side of the box where a light (waterproof professional video light, re-fuel® by DIGIPOWER) was inserted. The acrylic plate that was used to extend the surface area to match the box diameters was not touching the sides of the box, ensuring that the weight of the birds was always on the force plate. The force plate was connected to a laptop where the expended force was recorded continuously (Bertec Digital Acquire 4.0.11.407). The light was switched on after the force plate (Bertec Force Plate Version 1.0.0, BERTEC Corporation, Columbus, Ohio, USA) started recording. Output measurements were the force vectors of the horizontal (Fy and Fx) and vertical (Fz) forces expended.

In weeks 4 and 14, 20 birds from each aviary-housed treatment group and 10 birds from both *Conv* strains were tested in a startle test. All birds were tested in one day, with the treatment groups’ testing order changing with every flock, ensuring that the testing time was balanced between all groups. The experimental protocol was based on the procedure reported by Ross *et al*. [41]. Birds were collected (using convenience sampling) in their housing system and carried in crates to a testing room. To avoid a sampling bias for calm or bold birds, the lights were turned off during capturing as chickens are inactive in the dark. The experimenter could not be blinded to treatment as genetic strain and housing system were visually distinguishable. The testing room was dark, and crates were kept in the dark for a minimum of 15 min and a maximum of 30 min before testing commenced. The testing apparatus consisted of a box with a force plate as the base to record the expended force (see figure 2). Birds were tested individually. Each bird was placed in the box, facing a light (63 superbright LEDs, 50 Watt) that was enclosed in one wall (waterproof professional video light, re-fuel^®^ by DIGIPOWER, ^©^2018 MIZCO International, Inc. www.mizco.com.au). After the lid was closed, the force plate reading was zeroed, and the recording started. The light was switched on, and the startle amplitude was recorded as the maximum force exerted on the force plate. The bird was then returned to a different crate before the whole group was marked with wing tags to prevent further testing and returned to their housing system.

**Figure 3.**
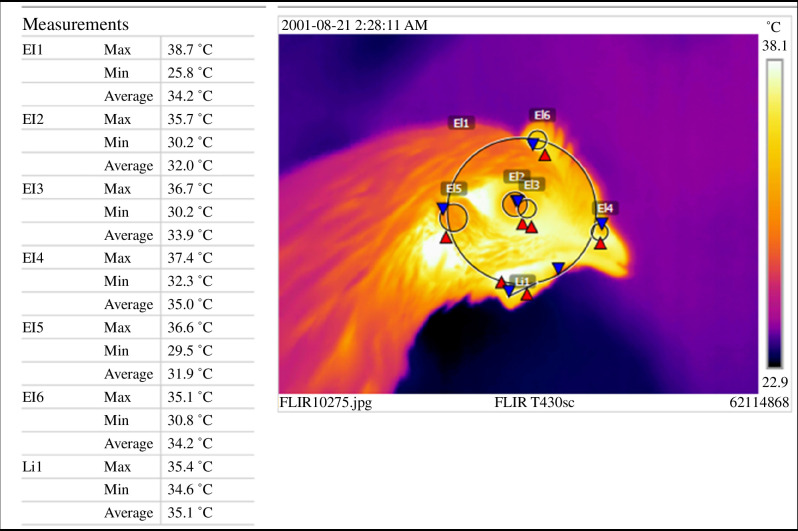
Example of a thermal measurement output for a pullet (camera: Flir T430 Thermal Imaging Camera, Hoskin Scientific Limited, Oakville, Ontario, Canada, software: FLIR Tools version 5.13.18031, 2002). Measurements inform on the maximum (Max), minimum (Min) and average temperature of six ellipses (El) and one line (Li) in degrees Celsius. ‘El1’ = head, ‘El2’ = eye, ‘El3’ = eye angle, ‘El4’ = nostril, ‘El5’ = ear, ‘El6’ = comb and ‘Li1’ = wattle. Locations were chosen based on [42].

An additional measure was added for flocks 3 and 4 to allow for autonomic response assessment. For this purpose, thermal images of the birds' heads were taken while manually restrained before placement in the box and immediately (within 10 s) after the test (see figure 3). The images were taken too long after the capture to serve as a true baseline (to avoid stress from individual marking before the startle), and they were taken to assess any immediate changes in temperature in response to the startle. Thermal images were analysed using a thermal analysis software (detailed in figure 3). Temperature measurements (min, max and average in degree Celsius) were taken for six (in chicks) or seven areas (in pullets) (based on [42]): eye, eye angle (beak-facing corner of the eye), ear, nostril, comb, whole head and wattle (pullets only). Eighty images were analysed twice for reliability assessment.

### 2.4. NA and NO test

In week 14, a combined NA-NO test was performed. Data collection was restricted during flocks 2 and 3 because of the global COVID-19 pandemic. Hence, fewer birds were tested in these flocks than in flocks 1 and 4 (flock 1: 208, flock 2: 120, flock 3: 144 and flock 4: 216). The testing order was balanced between housing systems and strains, and the testing took either four (flocks 1 and 4) or two days (flocks 2 and 3). A total of 96 birds per strain were tested from each aviary and 56 birds per strain from the *Conv* treatment.

**Figure 4.**
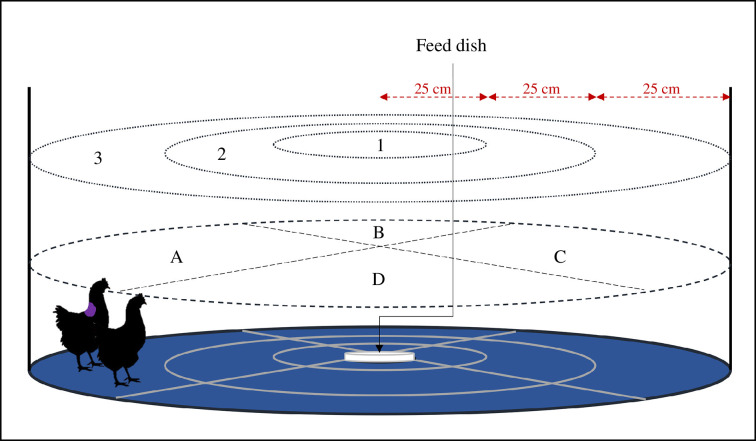
Testing arena for the NA:NO test and two identical arenas were used. The arenas visually split into zones; quadrants (*a–d*, dashed line) and rings (1–3, dotted line). Ring 1 had a diameter of 50 cm, ring 2 100 cm and ring 3 150 cm. At the centre of the arena was a white food dish with a standard-laying hen grower feed. Hens were placed in pairs into ring 3 and observed with an overhead camera (Sony HDR-CX405 HD Video Recording Handycam Camcorder). One hen was marked with livestock paint (KONK Livestock Marker) for individual recognition.

Two circular testing arenas located in an empty room were used with a diameter of 1.5 m each (see figure 4). They had dark blue plastic floors and were visually divided into zones using grey tape; four quadrants (A–D) and three rings. A white plastic plate with a standard feed was fixed to the centre of the arena using hook and loop tape (Velcro^®^ Brand) for easy removal and cleaning. The purpose of the dish was to encourage the pullets to remain in the centre of the arena after approaching it. A camera was mounted directly over each arena. For each round of tests, four pullets were selected (convenience sampling in the dark) in their housing systems, carried to the testing room and placed in a crate. After 10 min, two pullets were marked with paint (to help discriminate birds on camera), and then, testing began for each pair in a separate arena.

The test consisted of two phases, a novel arena (NA) phase and a novel object (NO) phase. At the start of the NA phase, pullets were placed in the arenas in pairs (one marked and one unmarked pullet) in zone C3. The experimenter left the room while the cameras recorded the test. After 10 min, the observer returned and, staying out of sight, slowly lowered a NO suspended from the ceiling into the centre. A multi-coloured cardboard tube originating from a kitchen towel roll was used as the NO. The test continued for five more minutes before the recording was stopped. Pullets were collected, marked to prevent re-testing and returned to their housing systems. Coded notes were shown to the cameras before each test to identify the pair in the arena. A single observer, blinded to housing treatment, watched all the videos. During the NA phase, the pullet position (zone) and alert behaviour (ethogram in table 1) were recorded at 1-min intervals on an individual basis. During the NO phase, the first reaction to the appearance of the NO was recorded as either avoidance (walking, running and jumping away within 5 s of the NO appearance) or stationary (no locomotion for 5 s or longer). The secondary response to the NO was assessed by recording the pullet position (zone) at 30-s intervals. During both phases, latency to approach the centre (NA test; scan samples every 1 min) or the NO (NO test; scan samples every 30 s) was defined as the first scan where the pullet had both feet in ring 1. Twenty videos were recoded to assess intra-observer reliability (IOR).

**Table 1.** Ethogram used to assess alert behaviour in the NA. Adapted from [43] and [44].

behaviour	definition
*not alert*	
exploring	the pullet is moving with her head facing down
ground pecking	the pullet is stationary while repeatedly touching her beak to the floor
feeding	the pullet is in ring 1 with her head over the feed dish
sitting	the pullet is stationary, on the ground, with her legs tucked under her
dustbathing	the pullet is stationary, on her side, raking her beak or feet on the floor. Her wings are either extended or tucked in
preening	the pullet is stationary, either sitting or standing with her beak moving in her feathers
*alert*	
alert standing	the pullet is stationary with her neck stretched and eyes open
alert walking	the pullet is locomoting with her neck stretched
attempting escape	the pullet is running or jumping towards the walls of the arena with her neck stretched; her wings can be used to assist in locomotion
vocalizing	the pullet is stationary or moving with her beak open, and vocalizations are audible

### 2.5. Data processing

Startle amplitude was calculated as the force in millinewton that was exerted based on the three force vectors (Fy, Fx and Fz; figure 2) provided by the force plate output. The force amplitude i was calculated as


$$h=\sqrt{x^{2}+y^{2}},$$

$$i=\sqrt{h^{2}+z^{2}},$$

where *h* is the hypotenuse between the two horizontal force vectors ***x*** and ***y*** [45]. To describe the direction of the vector, i was multiplied by +1 or −1 in accordance with the sign of F*z*. The startle amplitude was corrected for the body weight by dividing the signed i by the average bird weight of each treatment group, which was calculated by individually weighing 15% of each population at 14 WoA. The bodyweight uniformity for week 4 was 91.9% for browns and 93.5% for whites, and in week 14, 93.2% for browns and 94.9% for whites. Autonomic response to the startle was assessed by calculating the difference in the max temperature before and after the startle for each area of the head. The reliability of thermal measurements was calculated for each area separately, and only those with good reliability were used going forward (table 2). Wattle temperature measures were disregarded as not all pullets had grown their wattles by week 14.

**Table 2.** Intra-class correlation (ICC) for each temperature change was measured in 80 thermal images that were scored twice by the same observer. Wattle temperature was only assessed on 12 images, as not all pullets had grown their wattles yet. Acceptable values are indicated in bold.

	single score ICC	
	ICC	95% CI
mean head temp. change	0.3	0.1–0.5
max head temp. change^a^	**0.9**	0.8–0.9
max eye temp. change	0.3	0.1–0.5
max eye angle temp. change	0.6	0.4–0.7
max nostril temp. change^a^	**0.8**	0.7–0.9
max ear temp. change	0.04	−0.2–0.3
max comb temp. change^a^	**0.9**	0.9–1.0
max wattle temp. change	**1.0**	1.0–1.0

^a^ Measures selected to assess the validity of the startle amplitude.

The exploration of the NA was calculated by the number of scan samples in which the zone differed from the previous scan sample (zone_scan#_ ≠ zone_scan#-1_). Alert behaviour was the total number of scans where a pullet was seen to perform any alert behaviour according to the ethogram (table 1). Both the scan (timepoint: scan#) and whether a pullet entered ring 1 (approached the centre/NO) were recorded (binary: yes = 1, no = 0). The primary reaction to the NO was recorded as binary (avoidance: yes = 1, no = 0). IOR was good for all measures taken in the NA/NO test: zone changes kappa = 0.85, alert behaviour kappa = 0.72, latency to approach the centre or NO kappa = 0.88 and the reaction to the NO kappa = 1.

### 2.6. Statistical analyses

Statistical analyses were done in R and R Studio version 4.1.2 (2021 The R Foundation for Statistical Computing) using packages ‘car’, ‘lme4’, ‘emmeans’, ‘irr’, ‘survival’, ‘survminer’ and ‘oddsratio’.

The startle amplitude and autonomic response were assessed by fitting linear mixed-effect models (LMMs) with strain, housing, age and their interactions as fixed effects. Random effects were age nested in the flock for the startle amplitude and flock and testing order for the autonomic response. The nesting accounted for the repeated testing of birds from each flock at two ages. A more complex random effect structure that accounted for the age nested within the rearing group explained close to 0% variation and, thus, was removed from the model to improve the fit. Wald chi-square statistics were used to assess the influence of individual factors on data distribution with a significance level of *p* = 0.05. A larger chi-square (χ^2^) value indicates that the factor explains more of the data distribution. Significant interactions were analysed with a post hoc Tukey test with Bonferroni correction for multiple testing. Effect sizes were calculated using the ‘eff_size’ command from the package ‘emmeans’, version 1.9.0.

To assess the reliability of thermal measures, single-score intra-class correlations were calculated for each measured area (table 2). There were three reliable temperature measurements: comb, nostril and whole head. In order to assess the relationship between the startle amplitude and autonomic response, a post hoc analysis was done (only flocks 3 and 4) by fitting an LMM to predict startle amplitude (log-transformed). Fixed effects were comb temperature change, nostril temperature change, head temperature change, age, strain and their interactions with age nested in flock as a random effect. Collinearity between the temperature variables was excluded after calculating the variance inflation factors (VIF = comb: 1.09, nostril: 1.13 and head: 1.05), which indicated no correlation between the predictors. The nested random effect accounted for birds within a flock being from the same statistical units. Based on those results, the dataset was split by strain (observations: 279 browns and 280 white) and startle force correlated (paired Pearson correlation) with temperature changes.

An LMM was fitted to assess alert behaviour and exploration in the NA, and a generalized LMM (GLMM) was to assess initial avoidance of the NO. Housing, strain and their interaction were fixed effects, and a pair nested in housing nested in the flock was the random effect. The approach of the centre of the NA was assessed by fitting a Cox proportional hazards ratio (Coxph). The Coxph calculates the hazard ratio (HR), or the odds of a pullet entering ring 1 at a certain scan compared with the odds of another pullet entering ring 1 at the same scan. The Coxph is a right-censored survival analysis class model that combines time and a binary outcome (approach yes or no) in a single model.

Odds ratios were calculated to compare initial NO avoidance within factors that significantly affected the model fit. The approach of the NO was assessed by fitting a Coxph.

The goodness of fit for mixed models was evaluated by visually assessing the distribution of the residuals. Unweighted Cohen’s Kappa was calculated to assess IOR.

## 3. Results

### 3.1. Startle test

**Figure 5.**
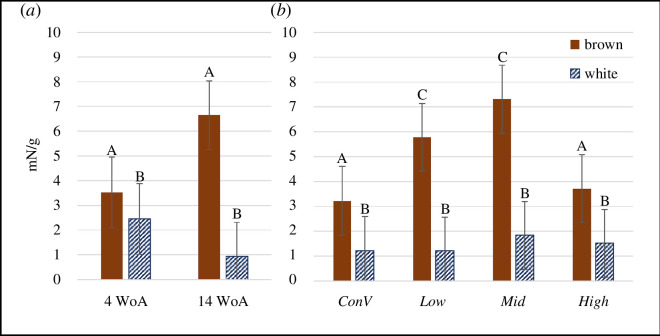
Startle amplitude (back-transformed LSMEAN ± s.e.) corrected for body weight. (*a*) The interaction of age and strain with week of age on the *x*-axis. (*b*) The interaction between the rearing environment (conventional cages (Conv) and aviary designs 1–3 (*Low*, *Mid* and *High*)) and strain. Brown birds are in brown and white birds in blue (patterned). Different letters indicate statistical differences (p < 0.05).

Startle amplitude (corrected for average population body weight) was explained by a strain-by-age interaction (χ^2^ = 98.5, *p* < 0.0001) and a housing-by-strain interaction (χ^2^ = 12.2, *p* = 0.007, figure 5). Brown birds had higher startle amplitudes (raw mean = 13.4 mN/g, max = 103.3 mN/g) than whites (raw mean = 3.1 mN/g, max = 59.3 mN/g) from all housing systems (brown versus white effect size [ES] ± standard errors and test statistic: *Conv*: ES = 0.73 ± 0.2, *t*-value [t] = 4.25; *Low*: ES = 1.22 ± 0.3, *t* = 9.88; *Mid*: ES = 1.09 ± 0.3, *t* = 8.78; *High*: ES = 0.68 ± 0.2, *t* = 5.43, *p* < 0.0001 for all comparisons). There were no housing effects within white birds. In browns, birds from *Conv* and *High* had lower startle amplitude than birds from *Low* (*Conv* versus *Low*: ES = −0.49 ± 0.2, *t* = 3.29, *p* = 0.006; *High* versus *Low*: ES = −0.37 ± 0.2, *t* = −3.01, *p* = 0.01) and *Mid* (*Conv* versus *Mid*: ES = −0.68 ± 0.2, *t* = −4.53, *p* < 0.0001; *High* versus *Mid*: ES = −0.56 ± 0.2, *t* = −4.49, *p* < 0.0001). There were no age effects for browns (*t* = 1.78, *p* = 0.19), and in whites, only a tendency for a higher startle amplitude in week 4 than 14 (ES = 0.84 ± 0.3, *t* = 2.82, *p* = 0.08). Browns had a higher startle amplitude than whites in both weeks 4 (ES = 0.24 ± 0.1, *t* = 2.39, *p* = 0.02) and 14 (ES = 1.62 ± 0.3, *t* = 17.57, *p* < 0.0001).

The temperature difference (before and after startle) in the comb was explained by an interaction of strain and age (χ^*2*^ = 5.7, *p* = 0.02) but not housing (χ^2^ = 6.5, *p* = 0.09). In week 14, white pullets had a larger drop in comb temperature than browns (mean = whites: −1.1 ± 0.1°C, browns: −0.4 ± 0.1°C, *t* = 3.95, *p* = 0.0001), but there was no difference between strains in week 4 (mean= whites: −0.5 ± 0.1°C, browns: −0.3 ± 0.1°C, *t* = 0.84, *p* = 0.4). Nostril temperature change was explained by age (χ^2^ = 29.1, *p* < 0.0001), with a larger drop in temperature in pullets than in chicks (mean = week 4: −0.7 ± 0.1°C, week 14: −1.4 ± 0.1°C, *t* = 5.29, *p* = 0.0001). Neither housing (χ^2^ = 1.5, *t* = 3, *p* = 0.7) nor strain (χ^2^ = 0.1, *t* = 1, *p* = 0.82) affected the change in nostril temperature. Head temperature change was not affected by housing (χ^2^ = 2.9, *p* = 0.41), strain (χ^2^ = 0.2, *p* = 0.69) or age (χ^2^ = 0.01, *p* = 0.92).

In order to assess the relationship between the startle amplitude and autonomic response, the temperature changes (before versus after the startle) in the comb, nostril and head were included in the model for Flocks 3 and 4. In this model, the startle amplitude was best predicted by an interaction of age and strain (χ^2^ = 57.0, *p* < 0.0001) and to a lesser degree by comb temperature change (χ^2^ = 4.0, *p* = 0.04) but not by nostril temperature change (χ^2^ = 0.1, *p* = 0.72) or head temperature change (χ^2^ = 1.3, *p* = 0.25). Strain explained a large variance with χ^2^ = 147.8. In brown birds, comb temperature change was negatively correlated with startle amplitude (higher amplitude with larger (more negative) temperature drop), albeit weakly (*r* = −0.1, *t* = −2.14, df = 269, *p* = 0.03), but there was no correlation with nostril (*r* = −0.1, *t* = −1.28, df = 276, *p* = 0.2) or head (*r* = 0.04, *t* = 0.73, df = 276, *p* = 0.47) temperature change. In white birds, no thermal measure correlated with startle amplitude (comb: *r* = 0.03, *t* = 0.65, df = 549, *p* = 0.52, nostril: *r* = −0.1, *t* = −0.81, df = 271, *p* = 0.42, head: *r* = 0.1, *t* = 0.81, df = 271, *p* = 0.42).

### 3.2. Novel arena

**Figure 6.**
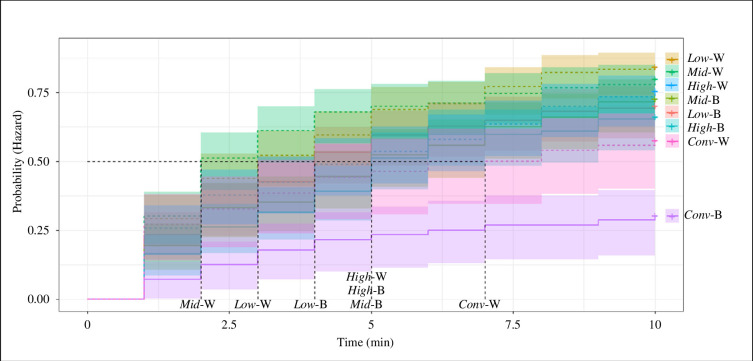
Probability of approaching the centre during the NA test. Dark lines are the mean probability and coloured bands around them indicate the 95% CIs. Solid lines represent brown (B) pullets and dotted lines whites (W). The treatment groups are indicated on the right (*Conv* = conventional cage and aviaries with *Low* = low, *Mid* = intermediate and *High* = high complexity). The dotted black lines indicate the time at which half of the treatment group would be expected to have approached the centre (hazard ≥ 0.5; Conv-B did not reach this point). The estimated HR of *Low* compared with *Conv* (*Low*-*Conv*) = 6.5, 95% CI = 2.97, 14.2; *Mid*-*Conv*: HR = 5.82, CI = 2.64, 12.8; *High*-*Conv*: HR = 4.4, CI = 2.01, 9.61. W *Conv*-B *Conv*: HR = 3.75, CI = 1.53, 9.20.

Exploration in the NA was explained by a housing effect (χ^2^ = 30.1, *p* < 0.0001, table 3) but not strain (χ^2^ = 0.001, *p* = 0.9). Pullets from *Conv* had fewer zone changes compared with any of the aviary groups (compared to *Low*: ES = −1.14 ± 0.4, *t* = 4.61, *p* = 0.004, *Mid*: ES = −1.23 ± 0.4, *t* = 4.94, *p* = 0.002, *High*: ES = −1.08 ± 0.4, *t* = 4.25, *p* = 0.006). Alert behaviour was explained by a housing (χ^2^ = 21.8, *p* < 0.0001) and a strain effect (χ^2^ = 16.4, *p* < 0.0001). Brown pullets had a higher count of alert behaviour than white pullets (ES = 0.68 ± 0.3, *t* = 3.80, *p* < 0.0001). Pullets from *Conv* were more often alert compared with aviary-reared pullets (*Low*: ES = 1.83 ± 0.6, *t* = −4.09, *p* = 0.01 *Mid*: ES = 1.57 ± 0.6, *t* = −3.49, *p* = 0.03, *High*: ES = 1.78 ± 0.6, *t* = −3.97, *p* = 0.01). The number and percentage of birds that approached the centre are given in table 4. Compared with *Conv* pullets, *Low* pullets had 6.5 times higher odds of approaching the centre of the arena (95% confidence interval (CI) = 3.0,14.2, figure 6), *Mid* had 5.8 times higher odds (CI = 2.6,12.8) and *High* pullets had 4.4 times higher odds (CI = 2.0,9.6). White birds from *Conv* had 3.75 times higher odds of approaching the centre than browns from *Conv* (CI = 1.5,9.2, figure 6).

**Table 3.** Least-squares means of the number of scans (out of 10 scan samples) for exploration and alert behaviour observed during the NA test. *p*-values are based on the Wald chi-square test statistic. Different letters indicate statistically different pairwise comparisons based on the post hoc analysis (p < 0.05). Last column: HR and 95% CI of approaching the centre of the arena. *p*-values indicate white birds compared to browns and pullets from each of the aviaries compared with conventional cage pullets.

	exploration	*p*‐value	alert behaviour	*p*‐value	approach centre HR	*p*‐value
	mean ± s.e.		mean ± s.e.			
strain		0.97		<0.0001		
brown	3.5 ± 0.2		7 ± 0.4^a^		—	
white	3.5 ± 0.2		6.0 ± 0.4^b^		1.2, 0.7–2.1	0.6
housing		<0.0001		<0.0001		
conv	2.2 ± 0.3^a^		8.3 ± 0.5^a^		—	
low	4 ± 0.3^b^		5.7 ± 0.5^b^		6.5, 3–14.2	<0.0001
mid	4.1 ± 0.3^b^		6.1 ± 0.5^b^		5.8, 2.6–12.8	<0.0001
high	3.8 ± 0.3^b^		5.8 ± 0.5^b^		4.4, 2.0–9.6	<0.0001
interaction		0.91		0.41		

**Table 4.** Number and percentage (%) of birds that approached the NA centre (in 10 min), actively avoided the NO (first reaction) and approached the NO (in 5 min) by housing and strain. *N* is the number of completed observations per group.

housing × strain	NA		NO		
	*N*	approached	*N*	avoidance	approached
conv	112	51	108	74	8
*%*		45.5		68.5	7.4
brown	56	17	56	30	4
white	56	34	52	44	4
low	192	149	190	175	38
*%*		77.6		92.1	20.0
brown	96	68	94	82	17
white	96	81	96	93	21
mid	192	150	176	154	39
*%*		78.1		87.5	22.2
brown	96	72	86	75	17
white	96	78	90	79	22
high	192	139	178	140	43
*%*		72.4		78.7	24.2
brown	96	65	90	63	17
white	96	74	88	77	26

### 3.3. NO test

The likelihood a bird would initially avoid the NO was affected by housing (χ^2^ = 34.5, *p* < 0.0001) and strain (χ^2^ = 18.8, *p* < 0.0001) but not their interaction (χ^2^ = 6.0, *p* = 0.11); see table 4 for the number and percentage of birds that actively avoided the NO at first. Pairwise comparisons with ORs where the 95% CI excluded ‘1’ were considered statistically different [46]. Pullets from *Low* had 6.4 times higher odds of avoiding the NO than *Conv* pullets and 3.6 times higher odds than *High* pullets (CI in table 5). *Mid* pullets had 3.3 times higher odds of avoiding the NO than *Conv* and 1.9 (CI = 1.1, 3.4) times higher odds than *High* pullets. Furthermore, *Low* pullets had higher odds of actively avoiding the NO at first appearance than *High* pullets (*Low*: OR = 3.6, CI = 1.6, 7.6). White pullets had 2.7 times higher odds of avoiding the NO than browns (CI = 1.7, 4.2).

**Table 5.** Odds ratio (OR, 95% CI) to avoid the NO at its initial appearance. Hazards ratio (HR, 95% CI) of approaching the NO within 5 min. Whites are compared with browns and aviary pullets compared with conventional cage pullets.

	1^st^ response	2^nd^ response	*p*‐value
	OR avoidance^a^	HR approach	HR
strain			
brown	—	—	
white	2.7, 1.7–4.2	1.4, 0.9–2.2	0.2
housing			
conv	—	—	
low	6.4, 3.2–13.3	3.0, 1.2–7.2	0.018
mid	3.3, 1.78–6.1	3.4, 1.4–8.4	0.007
high	1.8, 1–3.1	4.1, 1.7–10.0	0.002

^a^ OR are not assigned a *p*-value in R using the package ‘odds ratio’. Odds are considered different when the 95% CI does not include the value ‘1.0’.

The number and percentage of birds that subsequently approached the NO are given in table 4. *Low* pullets had 2.9 times (*p* = 0.02), *Mid* 3.4 times (*p* = 0.007) and *High* 4.1 times (*p* = 0.002) higher odds of approaching the NO than *Conv* pullets. White pullets were as likely to approach the NO within 5 min observed as brown pullets (HR = 1.4, *p* = 0.2).

## 4. Discussion

In this paper, we asked whether the degree of early housing complexity (minimal (*Conv*), low (*Low*), moderate (*Mid*) or high (*High*)), genetic strain (brown- versus white-feathered) or their interaction affected fear behaviour (type and intensity) in laying hen pullets in different fear-inducing situations. We hypothesized that increased early-life complexity would decrease indicators of fearfulness in the NA (more exploration and quicker approach of the centre/feed dish), towards the NO (lower odds of NO avoidance and quicker approach of NO/feed dish), the startle amplitude (lower amplitude) and the autonomic response (smaller drop in temperature) to the startle (fearfulness: *Conv* > *Low* > Mid > *High*). We further hypothesized that indicators of fear and fearfulness would differ between brown- and white-feathered birds.

Unexpectedly, brown birds from both the least (conventional cages) and the most complex environments had lower startle amplitudes than birds from the intermediate environmental treatments (*Conv*, *High* < *Low*, *Mid*), with no housing effect in whites. Housing did not affect the autonomic response to the startle stimulus in either strain. In support of our hypothesis, *Conv* pullets explored the NA less, were more alert and took longer to approach the centre of the NA compared to aviary-reared pullets. Unexpectedly, pullets from *Low* and *Mid* but not *High* were more likely to avoid the NO at first appearance than pullets from *Conv*. As predicted, aviary-reared pullets were more likely to approach the NO within 5 min than *Conv*-reared birds. As expected, there were strain effects on all behaviours measured. The startle amplitude range was smaller in whites than in browns, and whites had a stronger autonomic response in week 14. Compared to brown pullets, white pullets were less alert in the NA, more likely to avoid the NO at first sight, and when brown strains were reared in *Conv*, more likely to approach the NA centre within 10 min.

### 4.1. Startle test

The startle reflex is the immediate muscular response to an unexpected intense stimulus. It is thought to instigate flight to protect the individual from bodily harm [47]. The startle reflex test assesses the magnitude of the initial, unconscious startle and differs from the startle response test that often measures the recovery time after a startle [4,48]. The startle reflex amplitude is thought to reflect individual variation in affective state (reviewed by Grillon and Baas [47]). In a previous study, brown-feathered-laying hens (30 weeks old) housed in a preferred and enriched environment for two weeks had significantly reduced startle amplitudes compared with less enriched hens [41,49], which was interpreted as a more positive affective state in the enriched groups. In that same study, hens housed in enriched environments had lower baseline comb temperatures than non-enriched hens and tended to have a smaller decrease in comb temperature in response to an opening umbrella [49], though startle amplitude did not predict comb temperature decrease. The autonomic response is the rapid secondary response to the startle [50] and has been shown to increase with increasing startle amplitude in humans [51]. In the current paper, neither the startle amplitude nor the autonomic response to the startle yielded the expected results. Using a force plate to measure the expended force in reaction to a sudden light while taking thermal images before and after (methodology in this paper) did not appear to be a valid way to measure the startle reflex of the white strain of laying hens. Similar to Ross *et al*. [49], there was no correlation between physiological responses and startle amplitude in either strain. Additionally, the startle amplitude in the white pullets did not reflect the behavioural flight or freeze/immobility response recorded in the NO test. To the authors’ knowledge, the startle response test has never been used in white-feathered-laying hens, and their reaction could be influenced by factors that do not affect brown-feathered birds. A possible explanation could be that whites were more affected by the initial capture, transport and handling prior to testing than browns, as white strains have been shown to be more fearful of humans [20,26] and manual restraint [52]. If birds were already in a state of fear, it could have reduced any additional autonomic response to a non-detectable level, though startle amplitude is fear-potentiated and should have increased with fear level [53]. The comb temperature data used here were collected immediately before and after the birds were in the testing apparatus; hence, the first time point was following capture, crating and transport to the testing room. In other studies, baseline measures were taken before crating [49], and comb temp was sensitive enough to distinguish between handling methods [54]. In this study, logistics prevented us from collecting a true baseline measure without stressing the birds by adding an ID marker. The lack of a true baseline temperature measure before or immediately after capture made it impossible to verify whether the handling induced a stress response where no further increase in autonomic response was possible (plateau), because we were unable to compare the temperature before capture to that before the startle. In brown birds, on the other hand, startle amplitude did correlate with comb temperature, albeit weakly.

The startle amplitude of brown birds also corresponded with the behavioural reactivity (flight or freeze/immobility) to the appearance of the NO. *Conv* birds (low startle amplitude) took longer to investigate the NO after being startled by its appearance, suggesting a freezing reaction that is thought indicative of a high level of fearfulness [31]. *Low*, *Mid* (high startle amplitude) and *High* (low startle amplitude) birds recovered more quickly in the NO test than *Conv*, indicating a lower level of fearfulness [31]. The response to the startling visual stimulus (sudden light in a dark box) was apparently influenced by genetic strain and behavioural response profiles rather than the affective state. Future research should investigate the feasibility of recording eyeblinks as an alternative to expended force [47], as eyeblinks in chickens have been shown to be related to facial temperature changes in response to an acute stressor [55].

### 4.2. Fear behaviour

Both housing, genetic strain and their interaction affected responses in all three fear behaviour tests, if not in all measures collected. Fear behaviour follows a complex mechanism influenced by perception, experience and the current situation (figure 1). Stocking density acted as a potential confound in our study; however, it was decided that the benefits of a realistic representation of each commercially available environment (external validity, applicable to industry practices) outweighed the potential influences of stocking density. While high stocking density has been associated with increased anxiety in extremely overcrowded conditions [56], the conditions in that study were more than twice as dense as the highest stocking density in our study. A review of damaging behaviours and fearfulness in farm animals suggested that while fearfulness in poultry increases with the flock size, environmental complexity during rearing should mitigate that effect [38]. By combining results from all three tests, this paper provides insights into a hen’s initial behavioural response, including the consequent autonomic response and the eventual (conscious) behavioural response.

In both the startle response and the NO test, brown pullets reared in *Conv* had a different initial behavioural response than those raised in *Low* and *Mid*, adding to the existing pool of evidence that barren rearing leads to more fearful birds [35,36]. However, we also found differences between pullets from aviaries with different brooding compartment designs. Pullets from the most highly complex early-life environment were also less likely to flee from the NO than those from moderate or low complexity, suggesting that the degree of complexity might be key. Lack of active avoidance of the NO could be due to freezing (inhibition of activity, high fear level) but inactivity can also be explained by indifference (low fear level) [31]. Consistent housing effects were detected when pullets had time to assess and react to a new stimulus. Our results suggested that pullets from *Conv* had higher levels of fearfulness in both tests (NA and NO) compared to aviary-reared pullets, agreeing with differences reported between cage and cage-free-reared pullets [36]. The degree of aviary complexity did not affect the conscious behavioural response towards novel stimuli (approach/investigation). Our study provides evidence for a period of heightened developmental plasticity of fear behaviour during the early ontogeny of chickens, a mechanism that is well-established in mammals [57].

Brown pullets appeared more alert in the NA, and when reared in *Conv*, they were less likely to approach its centre, though both strains ambulated equally. We found strain differences in the initial reaction to a NO, which was likely perceived as a frightening stimulus. In this paper, white birds were fleers (active avoidance), and browns were freezers or indifferent (no active avoidance), which supports what was reported by Peixoto *et al.* [27] and Odén *et al.* [58], who reported whites to have a more active response to fear stimuli than browns. However, the exact opposite was reported by Nelson *et al*. [26]. They stated that browns actively avoid threatening stimuli (increased struggling in a struggle test, more wing flapping in an inversion test). At the same time, they found white hens used passive avoidance (less struggling and wing flapping, longer tonic immobility). The experimental approaches used in these different studies were quite different and not all fear tests might be appropriate to compare strains, for example, differences in activity levels could affect outcome measures. Additionally, while brown strains are phylogenetically removed from white strains, differences between distinct lines of brown hens or lines of white hens cannot be dismissed, which would add another layer of variation between studies. However, it appears that the perception of the stimulus could be different; what is frightening to a brown bird might not be as frightening to a white bird and vice versa [28]. We found no strain difference in the approach of the NO, which corresponds with some findings in the literature [27,59], though others reported browns to have shorter approach latencies [58], and a meta-analysis on strain differences in fear behaviour showed a lower approach rate in brown than white strains [28]. The same meta-analysis also suggested that strain differences in fear behaviour might be situation dependent [28]. Our results corroborate previous claims that different genetic strains might have different strategies to cope with specific stressors [28,60], as the results from the NA test did not match those from the NO test. Alternatively, these tests may measure different personality traits, not specific to the level of fearfulness (e.g. results from the open field and startle response test in African striped mice [4]).

## 5. Conclusion

In this paper, we asked whether the degree of early environmental complexity, genetic strain or their interaction affects fear behaviour (type and intensity) in laying hen pullets in a startle reflex test, an NA and an NO test. Overall, pullets from low or intermediately complex early-life environments had a more active fear response than those from barren environments who appeared most fearful, or those from high-complexity environments who appeared least fearful. White-feathered pullets had a more active fear response than brown pullets, though no assumptions can be made about their relative levels of fearfulness.

## Data Availability

The data supporting the presented findings and conclusions are openly accessible on Borealis [61].

## References

[1] Boissy A . 1995 Fear and fearfulness in animals. Q. Rev. Biol. **70** , 165–191. (10.1086/418981) 7610234

[2] Pamela Delarue EM , Kerr SE , Lee Rymer T . 2015 Habitat complexity, environmental change and personality: a tropical perspective. Behav. Processes **120** , 101–110. (10.1016/j.beproc.2015.09.006) 26386151

[3] Lecorps B , Kappel S , Weary DM , von Keyserlingk MAG . 2018 Dairy calves’ personality traits predict social proximity and response to an emotional challenge. Sci. Rep. **8** , 16350. (10.1038/s41598-018-34281-2) 30397225 PMC6218496

[4] Yuen CH , Schoepf I , Schradin C , Pillay N . 2017 Boldness: are open field and startle tests measuring the same personality trait? Anim. Behav. **128** , 143–151. (10.1016/j.anbehav.2017.04.009)

[5] Stankowich T , Blumstein DT . 2005 Fear in animals: a meta-analysis and review of risk assessment. Proc. Biol. Sci. **272** , 2627–2634. (10.1098/rspb.2005.3251) 16321785 PMC1559976

[6] Fox C , Merali Z , Harrison C . 2006 Therapeutic and protective effect of environmental enrichment against psychogenic and neurogenic stress. Behav. Brain Res. **175** , 1–8. (10.1016/j.bbr.2006.08.016) 16970997

[7] Tatemoto P , Valença-Silva G , Queiroz MR , Broom DM . 2021 Living with low environmental complexity increases fear indicators in Nile tilapia. Anim. Behav. **174** , 169–174. (10.1016/j.anbehav.2021.02.006)

[8] Appleby DL , Bradshaw JWS , Casey RA . 2002 Relationship between aggressive and avoidance behaviour by dogs and their experience in the first six months of life. Vet. Rec. **150** , 434–438. (10.1136/vr.150.14.434) 11993972

[9] Chamove AS . 1989 Cage design reduces emotionality in mice. Lab. Anim. **23** , 215–219. (10.1258/002367789780810608) 2761226

[10] Mora-Gallegos A , Fornaguera J . 2019 The effects of environmental enrichment and social isolation and their reversion on anxiety and fear conditioning. Behav. Processes **158** , 59–69. (10.1016/j.beproc.2018.10.022) 30389595

[11] Dietz L , Arnold AMK , Goerlich-Jansson VC , Vinke CM . 2018 The importance of early life experiences for the development of behavioural disorders in domestic dogs. Behaviour **155** , 83–114. (10.1163/1568539X-00003486)

[12] Xu W , Yao Q , Zhang W , Zhang F , Li H , Xu R , Li C , Zhang B . 2021 Environmental complexity during early life shapes average behavior in adulthood. Behav. Ecol. **32** , 105–113. (10.1093/beheco/araa108)

[13] Walker M , Duggan G , Roulston N , Van Slack A , Mason G . 2012 Negative affective states and their effects on morbidity, mortality and longevity. Anim. Welf. **21** , 497–509. (10.7120/09627286.21.4.497)

[14] Jones RB . 1996 Fear and adaptability in poultry: insights, implications and imperatives. Worlds Poult. Sci. J. **52** , 131–174. (10.1079/WPS19960013)

[15] Brantsæter M , Tahamtani FM , Nordgreen J , Sandberg E , Hansen TB , Rodenburg TB , Moe RO , Janczak AM . 2017 Access to litter during rearing and environmental enrichment during production reduce fearfulness in adult laying hens. Appl. Anim. Behav. Sci. **189** , 49–56. (10.1016/j.applanim.2017.01.008)

[16] Winter J , Toscano MJ , Stratmann A . 2021 Piling behaviour in Swiss layer flocks: description and related factors. Appl. Anim. Behav. Sci. **236** , 105272. (10.1016/j.applanim.2021.105272)

[17] Toscano MJ , Dunn IC , Christensen JP , Petow S , Kittelsen K , Ulrich R . 2020 Explanations for keel bone fractures in laying hens: are there explanations in addition to elevated egg production? Poult. Sci. **99** , 4183–4194. (10.1016/j.psj.2020.05.035) 32867962 PMC7597989

[18] Harlander-Matauschek A , Rodenburg TB , Sandilands V , Tobalske BW , Toscano MJ . 2015 Causes of keel bone damage and their solutions in laying hens. Worlds Poult. Sci. J. **71** , 461–472. (10.1017/S0043933915002135)

[19] de Haas EN , Bolhuis JE , de Jong IC , Kemp B , Janczak AM , Rodenburg TB . 2014 Predicting feather damage in laying hens during the laying period. Is it the past or is it the present? Appl. Anim. Behav. Sci. **160** , 75–85. (10.1016/j.applanim.2014.08.009)

[20] de Haas EN , Kemp B , Bolhuis JE , Groothuis T , Rodenburg TB . 2013 Fear, stress, and feather pecking in commercial white and brown laying hen parent-stock flocks and their relationships with production parameters. Poult. Sci. **92** , 2259–2269. (10.3382/ps.2012-02996) 23960107

[21] van Staaveren N , Harlander A . 2020 Cause and prevention of injurious pecking in chickens. In Understanding the behaviour and improving the welfare of chickens, pp. 509–566, Burleigh Dodds Science Publishing. (10.19103/AS.2020.0078)

[22] Agnvall B , Katajamaa R , Altimiras J , Jensen P . 2015 Is domestication driven by reduced fear of humans? Boldness, metabolism and serotonin levels in divergently selected red junglefowl (Gallus gallus). Biol. Lett. **11** , 20150509. (10.1098/rsbl.2015.0509) 26382075 PMC4614427

[23] Storengen LM , Lingaas F . 2015 Noise sensitivity in 17 dog breeds: prevalence, breed risk and correlation with fear in other situations. Appl. Anim. Behav. Sci. **171** , 152–160. (10.1016/j.applanim.2015.08.020)

[24] Romeyer A , Bouissou MF . 1992 Assessment of fear reactions in domestic sheep, and influence of breed and rearing conditions. Appl. Anim. Behav. Sci. **34** , 93–119. (10.1016/S0168-1591(05)80060-7)

[25] Simpson J , Kelly JP . 2011 The impact of environmental enrichment in laboratory rats—behavioural and neurochemical aspects. Behav. Brain. Res. **222** , 246–264. (10.1016/j.bbr.2011.04.002) 21504762

[26] Nelson JR , Settar P , Berger E , Wolc A , O’Sullivan N , Archer GS . 2020 Brown and white egg-layer strain differences in fearfulness and stress measures. Appl. Anim. Behav. Sci. **231** , 105087. (10.1016/j.applanim.2020.105087)

[27] Peixoto MRLV , Karrow NA , Newman A , Head J , Widowski TM . 2021 Effects of acute stressors experienced by five strains of layer breeders on measures of stress and fear in their offspring. Physiol. Behav. **228** , 113185. (10.1016/j.physbeh.2020.113185) 32980386

[28] Rentsch AK , Ellis JL , Widowski TM . 2023 Fearfulness in commercial laying hens: a meta-analysis comparing brown and white egg layers. Poult. Sci. **102** , 102664. (10.1016/j.psj.2023.102664) 37058921 PMC10123257

[29] Lyimo CM , Weigend A , Msoffe PL , Eding H , Simianer H , Weigend S . 2014 Global diversity and genetic contributions of chicken populations from African, Asian and European regions. Anim. Genet. **45** , 836–848. (10.1111/age.12230) 25315897

[30] Malomane DK , Simianer H , Weigend A , Reimer C , Schmitt AO , Weigend S . 2019 The SYNBREED chicken diversity panel: a global resource to assess chicken diversity at high genomic resolution. BMC Genomics. **20** , 345. (10.1186/s12864-019-5727-9) 31064348 PMC6505202

[31] Jones RB . 1987 The assessment of fear in the domestic fowl. In Cognitive aspects of social behaviour in the domestic fowl (eds R Zayan , IJH Duncan ), pp. 40–81, Amsterdam, The Netherlands: Elsevier Science Publishers B.V.

[32] Miller KA , Mench JA . 2005 The differential effects of four types of environmental enrichment on the activity budgets, fearfulness, and social proximity preference of Japanese quail. Appl. Anim. Behav. Sci. **95** , 169–187. (10.1016/j.applanim.2005.04.012)

[33] Meehan CL , Mench JA . 2002 Environmental enrichment affects the fear and exploratory responses to novelty of young Amazon parrots. Appl. Anim. Behav. Sci. **79** , 75–88. (10.1016/S0168-1591(02)00118-1)

[34] Van de Weerd HA , Aarsen EL , Mulder A , Kruitwagen C , Hendriksen CFM , Baumans V . 2002 Effects of environmental enrichment for mice: variation in experimental results. J. Appl. Anim. Welf. Sci. **5** , 87–109. (10.1207/S15327604JAWS0502_01) 12738579

[35] Brantsæter M , Nordgreen J , Rodenburg TB , Tahamtani FM , Popova A , Janczak AM . 2016 Exposure to increased environmental complexity during rearing reduces fearfulness and increases use of three-dimensional space in laying hens (Gallus gallus domesticus). Front. Vet. Sci. **3** , 1–10. (10.3389/fvets.2016.00014) 26973843 PMC4770049

[36] Brantsæter M , Tahamtani FM , Moe RO , Hansen TB , Orritt R , Nicol C , Janczak AM . 2016 Rearing laying hens in aviaries reduces fearfulness following transfer to furnished cages. Front. Vet. Sci. **3** , 13. (10.3389/fvets.2016.00013) 26955634 PMC4767898

[37] Campbell DLM , de Haas EN , Lee C . 2019 A review of environmental enrichment for laying hens during rearing in relation to their behavioral and physiological development. Poult. Sci. **98** , 9–28. (10.3382/ps/pey319) 30107615 PMC6347129

[38] Rodenburg TB , Koene P . 2007 The impact of group size on damaging behaviours, aggression, fear and stress in farm animals. Appl. Anim. Behav. Sci. **103** , 205–214. (10.1016/j.applanim.2006.05.024)

[39] NFACC . 2017 Code of practice for the care and handling of pullets and laying hens. National Farm Animal Care Council.

[40] Rentsch AK , Harlander A , Siegford JM , Vitienes I , Willie BM , Widowski TM . 2023 Rearing laying hens: the effect of aviary design and genetic strain on pullet exercise and perching behavior. Front. Anim. Sci. **4** . (10.3389/fanim.2023.1176702)

[41] Ross M , Garland A , Harlander-Matauschek A , Kitchenham L , Mason G . 2019 Welfare-improving enrichments greatly reduce hens’ startle responses, despite little change in judgment bias. Sci. Rep. **9** , 1–14. (10.1038/s41598-019-48351-6) 31417122 PMC6695442

[42] Moe RO , Bohlin J , Flø A , Vasdal G , Stubsjøen SM . 2017 Hot chicks, cold feet. Physiol. Behav. **179** , 42–48. (10.1016/j.physbeh.2017.05.025) 28528892

[43] Håkansson J , Jensen P . 2005 Behavioural and morphological variation between captive populations of red junglefowl (Gallus gallus)—possible implications for conservation. Biol. Conserv. **122** , 431–439. (10.1016/j.biocon.2004.09.004)

[44] Tahamtani FM , Hansen TB , Orritt R , Nicol C , Moe RO , Janczak AM . 2014 Does rearing laying hens in aviaries adversely affect long-term welfare following transfer to furnished cages? PLoS One **9** , e107357. (10.1371/journal.pone.0107357) 25229879 PMC4167866

[45] Ross M . 2018 Hens with benefits: enrichments’ effects on resilience in laying hens. University of Guelph. See http://hdl.handle.net/10214/14657 (accessed 17 December 2018)

[46] Szumilas M . 2010 Explaining odds ratios. J. Can. Acad. Child Adolesc. Psychiatry **19** , 227–229. (10.1136/bmj.c4414) 20842279 PMC2938757

[47] Grillon C , Baas J . 2003 A review of the modulation of the startle reflex by affective states and its application in psychiatry. Clin. Neurophysiol. **114** , 1557–1579. (10.1016/s1388-2457(03)00202-5) 12948786

[48] Nielsen BL . 2020 Asking animals: an introduction toanimal behaviour testing. Wallingford, UK: CABI. (10.1079/9781789240603.0000)

[49] Ross M , Rausch Q , Vandenberg B , Mason G . 2020 Hens with benefits: can environmental enrichment make chickens more resilient to stress? Physiol. Behav. **226** , 113077. (10.1016/j.physbeh.2020.113077) 32738316

[50] Martin WL , Murray PS , Bates PR , Lee PSY . 2015 Fear-potentiated startle: a review from an aviation perspective. Int. J. Aviat. Psychol. **25** , 97–107. (10.1080/10508414.2015.1128293)

[51] Globisch J , Hamm AO , Esteves F , Ohman A . 1999 Fear appears fast: temporal course of startle reflex potentiation in animal fearful subjects. Psychophysiology **36** , 66–75. (10.1017/s0048577299970634) 10098381

[52] Uitdehaag KA , Rodenburg TB , van Hierden YM , Bolhuis JE , Toscano MJ , Nicol CJ , Komen J . 2008 Effects of mixed housing of birds from two genetic lines of laying hens on open field and manual restraint responses. Behav. Processes **79** , 13–18. (10.1016/j.beproc.2008.04.004) 18511218

[53] Davis M. 2007 Neural systems involved in fear and anxiety based on the fear-potentiated startle test. In Neurobiology of learning and memory, pp. 381–425. Burlington, VT: Elsevier. (10.1016/B978-012372540-0/50013-3)

[54] Herborn KA , Graves JL , Jerem P , Evans NP , Nager R , McCafferty DJ , McKeegan DEF . 2015 Skin temperature reveals the intensity of acute stress. Physiol. Behav. **152** , 225–230. (10.1016/j.physbeh.2015.09.032) 26434785 PMC4664114

[55] Pijpers N , van den Heuvel H , Duncan IH , Yorzinski J , Neethirajan S . Understanding chicks’ emotions: are eye blinks & facial temperatures reliable indicators? In bioRxiv pp. 338–368 See 10.1101/2022.01.31.478468

[56] von Eugen K , Nordquist RE , Zeinstra E , van der Staay F . 2019 Stocking density affects stress and anxious behavior in the laying hen chick during rearing. Animals (Basel). **9** , 1–18. (10.3390/ani9020053) PMC6406350 30744165

[57] Wiedenmayer CP . 2009 Plasticity of defensive behavior and fear in early development. Neurosci. Biobehav. Rev. **33** , 432–441. (10.1016/j.neubiorev.2008.11.004) 19073211 PMC2671008

[58] Odén K , Keeling LJ , Algers B . 2002 Behaviour of laying hens in two types of aviary systems on 25 commercial farms in Sweden. Br. Poult. Sci. **43** , 169–181. (10.1080/00071660120121364) 12047079

[59] Fraisse F , Cockrem JF . 2006 Corticosterone and fear behaviour in white and brown caged laying hens. Br. Poult. Sci. **47** , 110–119. (10.1080/00071660600610534) 16641020

[60] Pusch EA , Bentz AB , Becker DJ , Navara KJ . 2018 Behavioral phenotype predicts physiological responses to chronic stress in proactive and reactive birds. Gen. Comp. Endocrinol. **255** , 71–77. (10.1016/j.ygcen.2017.10.008) 29051076

[61] Rentsch A , Harlander A , Niel L , Siegford J , Widowski T . 2023 Data from: Raising laying hens: housing complexity and genetic strain affect startle and behavioural response to fear stimuli. Borealis. (10.5683/SP3/3RJVMY) PMC10951723 38511084

